# Inference of Type-Specific HPV Transmissibility, Progression and Clearance Rates: A Mathematical Modelling Approach

**DOI:** 10.1371/journal.pone.0049614

**Published:** 2012-11-21

**Authors:** Helen C. Johnson, K. Miriam Elfström, W. John Edmunds

**Affiliations:** 1 Department of Infectious Disease Epidemiology/Centre for the Mathematical Modelling of Infectious Diseases, London School of Hygiene and Tropical Medicine, London, United Kingdom; 2 Department of Medical Epidemiology and Biostatistics, Karolinska Institutet, Stockholm, Sweden; National University of Singapore, Singapore

## Abstract

Quantifying rates governing the clearance of Human Papillomavirus (HPV) and its progression to clinical disease, together with viral transmissibility and the duration of naturally-acquired immunity, is essential in estimating the impact of vaccination programmes and screening or testing regimes. However, the complex natural history of HPV makes this difficult. We infer the viral transmissibility, rate of waning natural immunity and rates of progression and clearance of infection of 13 high-risk and 2 non-oncogenic HPV types, making use of a number of rich datasets from Sweden. Estimates of viral transmissibility, clearance of initial infection and waning immunity were derived in a Bayesian framework by fitting a susceptible-infectious-recovered-susceptible (SIRS) transmission model to age- and type-specific HPV prevalence data from both a cross-sectional study and a randomised controlled trial (RCT) of primary HPV screening. The models fitted well, but over-estimated the prevalence of four high-risk types with respect to the data. Three of these types (HPV-33, -35 and -58) are among the most closely related phylogenetically to the most prevalent HPV-16. The fourth (HPV-45) is the most closely related to HPV-18; the second most prevalent type. We suggest that this may be an indicator of cross-immunity. Rates of progression and clearance of clinical lesions were additionally estimated from longitudinal data gathered as part of the same RCT. Our estimates of progression and clearance rates are consistent with the findings of survival analysis studies and we extend the literature by estimating progression and clearance rates for non-16 and non-18 high-risk types. We anticipate that such type-specific estimates will be useful in the parameterisation of further models and in developing our understanding of HPV natural history.

## Introduction

Persistent infection with high-risk human papillomavirus (HPV) has been shown to be the necessary precursor of progression to cervical cancer [1], [2], [3]. The introduction of two prophylactic vaccines and the advent of new screening technologies have led to a surge in the number of HPV modelling studies. Such analysis can give insight into, for example, the epidemiological impact of combined HPV testing and conventional Pap smear screening; the marginal effect of screening in vaccinated populations or the effect of population-level behavioural heterogeneity on the impact of a screening or vaccination programme. The combined usefulness and challenge of modelling is the understanding and quantification of uncertainty. HPV infection has a particularly complex natural history: an initial infection may lead to the development of clinical lesions which either progress to a more severe grade of neoplasia or clear spontaneously. The more advanced the lesion, the lower the chance of viral clearance [4], [5]. Also, the risk of progression to high-grade lesions, and then subsequently to carcinoma, is higher for some HPV types than others: 70% of cervical cancers are associated with either HPV-16 or HPV-18 [6], [7], [8], [9]. Consequently, HPV modellers must make judicious choices of model structure and prior ranges for parameters determining e.g. the transmissibility of infection, rates of progression and clearance of clinical lesions and the rate of waning natural immunity following infection. Some HPV models of the early vaccine era were parameterised with one pre-specified set of parameters, assessing the sensitivity of model outcomes to each parameter in turn [10], [11], [12]. This approach limits the understanding of the interaction between input parameters. Both these studies and others [13], [14] derived HPV natural history parameters from cohort studies in the epidemiological literature [2], [15], [16], [17]. However, such clinical studies are limited by wide intervals between follow-up appointments (which may allow multiple events to occur), unclear distinctions between the diagnosis of neoplastic grades and, in the case of estimating progression rates to cancer, ethical considerations. For this reason, there has been a move towards inferring HPV natural history parameters by fitting mathematical (mechanistic) models of HPV transmission and progression to point prevalence data on HPV infection and disease states. By so doing, it is possible to incorporate prior knowledge about the progression of infection and the protocols for screening and treatment in the study population. Early work on such inference of HPV-related parameters relied on obtaining a visually good fit between model projections and observed data [13], [18], [19]. Such an approach gives insight into model structures and parameter values that will yield a good fit to data but these are multiple and various [5]. Recently Kim et al. [20], Van de Velde [21] and Jit et al. [5] have evaluated the goodness of fit between model output and observed data for a wide range of possible model structures and systematically chosen parameter values and Bogaards et al. [22] have applied Markov Chain Monte Carlo (MCMC) techniques to characterise posterior distributions for the rates of transmissibility and waning natural immunity of 14 high-risk HPV types. These developments have allowed the beginnings of quantitative understanding of both the uncertainty and sensitivity of HPV natural history parameters. Such an insight is crucial to confident predictions of vaccination or screening programme effectiveness.

We have aimed to infer the viral transmissibility, rate of waning natural immunity and rates of progression and clearance of infection of 13 high-risk HPV types and for HPV-6 and -11, making use of two rich epidemiological datasets and a comprehensive survey of sexual activity from Sweden. Estimates of viral transmissibility, clearance of initial infection and waning immunity were derived in a Bayesian framework by fitting a susceptible-infectious-recovered-susceptible (SIRS) transmission model to age- and type-specific HPV prevalence data from both a cross-sectional study and a randomised controlled trial (RCT) of primary HPV screening. Rates of progression and clearance of clinical lesions were estimated from longitudinal data gathered as part of the same RCT. Where possible we compare our estimates of these parameters with those derived from similar studies.

## Materials and Methods

### HPV Transmission Model

We developed a deterministic model of heterosexual partnership formation, mono-type HPV transmission and the potential progression to cervical cancer for each of 13 high-risk HPV types (16, 18, 31, 33, 35, 39, 45, 51, 52, 56, 58, 59 and 66) and for HPV-6 and -11. We model an age- and sexual activity- structured population with two sexual activity groups for each of three age brackets (14–29; 30–60 and 60+) where the size of the high sexual activity group is a fixed proportion (15%) of the overall population, regardless of age. Contact rates for each of these six age/sexual activity groups were derived from the Swedish sexual activity data gathered as part of the *Sex in Sweden* study [23], see *Parameterisation and Data*. These data also informed the boundaries of the age brackets.

**Figure 1:**
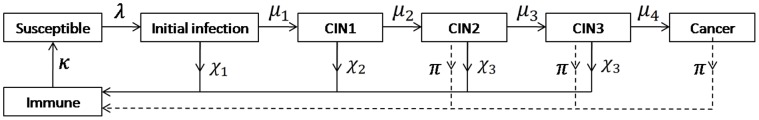
The *Susceptible-Infectious-Recovered-Susceptible* (SIRS) model of high-risk Human Papillomavirus (HPV) transmission and potential consequent progression to cervical cancer. Susceptible individuals become infected at a rate proportional to the force of infection λ. Following infection, they may progress in a stepwise fashion to neoplastic lesions of increasing severity (CIN1, CIN2, CIN3) and then to cancer (with rates ). Alternatively, they may spontaneously clear the infection from any pre-cancerous stage (with rates ). High-grade clinical lesions (CIN2 and CIN3) may be identified by cytological screening and successfully treated (at a rate π). Following viral clearance or successful treatment of lesions, women retain an immunity to re-infection with the same HPV type. This immunity wanes at rate κ, precipitating a return to the Susceptible compartment.

We assumed an SIRS structure for HPV infection in both men and women, allowing for an SIR structure in the limit where the rate of waning natural immunity, estimated within this analysis, tended to zero. The infected (I) compartment for females was divided into five sub-compartments, corresponding to the progression of HPV infection to disease: Initial infection, CIN1, CIN2, CIN3 and Cancer (where CIN stands for *Cervical Intraepithelial Neoplasia)*. Women with an initial infection may either progress to CIN1-type lesions at a rate of or naturally clear the virus at a rate of 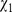. The rate of clearance of infection in males was considered equal to the rate of natural clearance in women [24]. Women with CIN-type lesions either progress to higher-grade lesions and then to cancer in a stepwise fashion (at a rate dependent on stage, 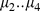) or clear the virus at rate dependent on stage (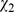 for CIN1 and 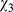 for CIN2/3 lesions). Note that the clearance rate for CIN2 and CIN3 lesions is assumed to be the same; this allows for the scarce data available on infections of these grades and uncertainty in the clinical classification between CIN2 and CIN3. We did not consider spontaneous clearance at the cancer stage. The model structure for high-risk HPV transmission and progression is shown in Figure 1. HPV-6 and HPV-11 and all HPV types in men were modelled using a simple SIRS structure without sub-division by progression stage. Equations for the partial differential equations employed are given in the Supplementary Material (S1).

It was assumed that only the initial infection with HPV and lower-grade cervical disease (CIN1 and CIN2-type lesions) are infectious. Also, the force of infection was calculated assuming that viral transmissibility 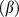 is independent of gender, age, sexual activity class and stage of infection. Thus the force of infection acting on females is:
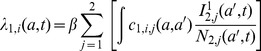
where state variables take the form



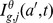
with


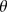
: indicating sub-division of the infected stage, 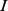. In the case of males this takes only the value 1.


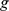
: gender (1 =  females, 2 = males)


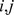
: sexual activity groups of the subject and his/her partner respectively


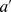
: used to denote the age of people of the opposite gender, 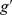


The state variables used in the model are summarised in the Supplementary Material.


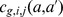
 is the annual partnership formation rate, described below

and
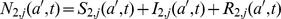
is the total population of males, 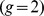.

The force of infection acting on males depends on the number of females in each of the three infectious 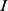 compartments:
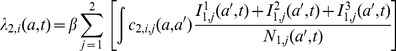
where the total population of females



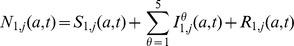



At demographic equilibrium, 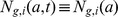 and therefore henceforth we disregard the temporal dependence of population size.

**Table 1:** The estimated annual rates of new partner acquisition for males and females in each of the three age and two sexual activity groups.

	Males		Females	
Age	Low sexual activity	High sexual activity	Low sexual activity	High sexual activity
14–29	0.54	4.11	0.25	2.89
30–59	0.05	0.64	0.07	1.47
60+	0.01	0.21	0.02	0.11

Mixing in the model is determined on a proportionate random basis i.e. without accounting for either assortativity on the basis of either age or sexual activity group. In order to balance the number of partnerships between males and females we assume that females adjust their behaviour completely to the number of partnerships sought by males. This leads to the simplistic conclusion that the overall rate of partnerships formed between females of age 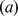 and sexual activity group with males of age 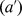 sexual activity group is:
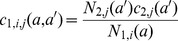



Cervical screening was incorporated into the model according to the Swedish screening protocol. In Sweden, women are screened at 3-yearly intervals between the ages of 23 and 50 and at 5-yearly intervals between 51 and 60. Uptake is 79% (J. Dillner, personal communication) and we made the limiting assumption that this rate is independent of sexual behaviour. Treatment is provided for screened women with CIN2+ lesions and cancer, resulting in the clearance of infection; the combined rate for screening and subsequent successful treatment is π.

We assumed that the rates of waning immunity and clearance of initial infection are independent of age, gender, sexual activity and personal history of HPV infection.

### Parameterisation/Data

Sexual behaviour in the model (e.g. partnership formation rates) was parameterised using data from a comprehensive Swedish sexual activity survey (n = 2810). Complete details of the survey have been published in ‘*Sex in Sweden*’ [23].

**Table 2:** The assumed equivalence between histological and cytological test results used in estimating rates of disease progression and clearance.

*Histological test*	*Cytological test*
Within normal limits	Within normal limits
	Koilocytosis
CIN1	ASCUS/LSIL
CIN2/3	HSIL

### Estimation of Partner Acquisition Rates

As described above, the rate of partner acquisition, 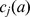, defined as the number of new partners per year, varies according to age 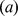 and sexual activity group 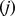.These rates were estimated fitting a mixture of Poisson distributions to data using an MCMC routine. The data used were the annual rates of new partners, stratified by gender and age, from the Swedish sexual activity survey (Lewin et al., 2000). The rationale for deriving partner acquisition rates is outlined below:

Given a certain annual rate of partner acquisition 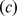 for a subpopulation, we would expect the number of new partners per year 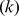 to be Poisson-distributed:



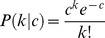



We consider males and females, and each age group, separately. Defining *a priori*, the number 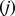 and proportions 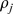 of each sexual activity group, we can describe the overall probability of having a certain number of partners 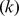 as a mixture of Poisson distributions:



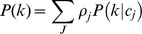
where 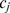 is the partner acquisition rate for sexual activity group 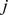.

Since we have data on the number of new partners per year, stratified by age 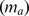, we can estimate the rates 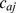
with an MCMC routine, maximising the likelihood



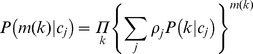
again considering each age group separately.

In the parameterisation of this model, we considered two sexual activity groups, the high sexual activity group, 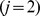, accounting for 15% of the population. We set the condition that 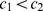.

The proposal densities were standard Normal distributions with standard deviation dependent on the sexual activity group:
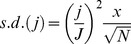
where 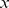 is a scaling constant and 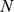 is the total population size.

Since the higher sexual activity group has a smaller population, it is more difficult to fit the likelihood than for the larger low sexual activity group. For this reason we specified a prior function of exponential form: 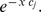


The consequence of this was to penalise higher contact rates, essentially attempting to describe the data with rates as low as possible. The resultant estimated annual rates of new partner acquisition are shown in Table 1.

**Figure 2:**
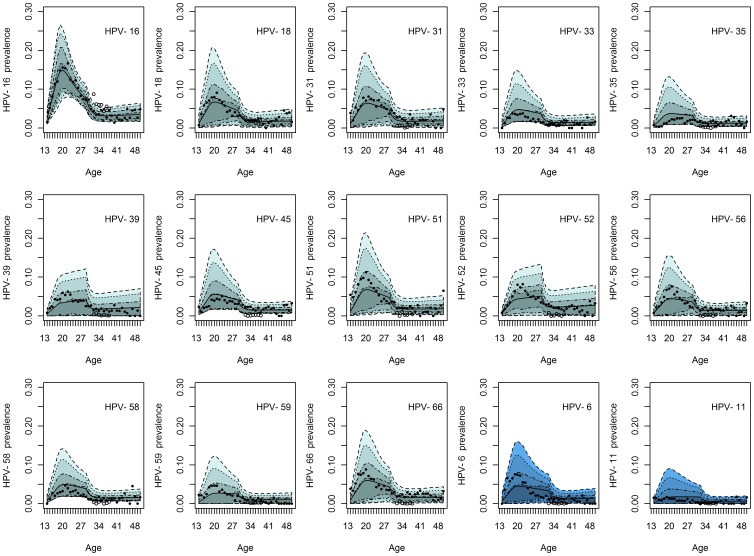
The predicted endemic prevalence of type-specific HPV infection in sexually active women and the observed prevalence in both women attending voluntary Chlamydia screening (solid dots) and those tested at baseline in the Swedescreen study (hollow dots). The median predicted prevalence for each type is represented by the solid curve and the turquoise bands represent the 50%, 70%, 90% and 95% posterior intervals.

### Data

In order to estimate the per-partnership viral transmissibility 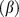, annual rate of clearance of initial infection 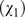 and annual rate of waning natural immunity 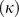, the model was fitted to both data from the Swedescreen trial and also data from the Swedish voluntary Chlamydia screening programme.

Swedescreen is a prospective randomised controlled trial of 12,527 women, aged 32–38, attending population-based invitational cervical screening in Sweden. Women were allocated in a 1∶1 ratio to either receive an HPV test as well as a cytology test (intervention) or a cytology test alone (control). HPV positive women were invited for a second HPV test at least twelve months later and women with persistent infections were invited to colposcopy. Women were then followed with comprehensive registry-based follow-up. Further details of the study have been published elsewhere [25], [26], [27].

**Table 3:** The estimated rates of progression and clearance of CIN1-type lesions in four high-risk HPV types.

ANNUAL RATE	16	18	31	33	OHR
Progression of HPV to CIN1	0.026 (0.007, 0.045)	0.058 (0.000, 0.179)	0.039 (0.008, 0.073)	0.046 (0.000, 0.147)	0.020 (0.004, 0.040)
Progression of CIN1 to CIN2	0.042 (0.010, 0.087)	0.111 (0.000, 0.255)	0.069 (0.000, 0.182)	0.182 (0.000, 0.406)	0.111 (0.048, 0.192)
Progression of CIN2 to CIN3	0.124 (0.024, 0.235)	–	0.136 (0.024, 0.240)	0.105 (0.000, 0.357)	0.169 (0.072, 0.276)
Progression of CIN3 to Cancer	0.026 (0.000, 0.080)	–	–	–	–
Clearance of CIN1	1.468 (0.893, 2.043)	1.257 (0.286, 2.197)	1.136 (0.531, 1.672)	1.386 (0.365, 2.197)	1.212 (0.766, 1.645)
Clearance of CIN2+	1.082 (0.710, 1.438)	0.884 (0.148, 1.695)	0.496 (0.191, 0.833)	0.788 (0.201, 1.299)	0.606 (0.393, 0.822)

To facilitate comparison with other studies, the grouping OHR includes types -31 and -33. Results presented are the median and 95% adjusted bootstrap confidence interval. There were insufficient data available for non-16 types to be able to infer the progression rates of CIN3 to cancer and, in the case of HPV-18, the rate of progression from CIN2 to CIN3.

The data from the Swedish voluntary Chlamydia screening programme provided information on the prevalence of each of the 13 high-risk HPV types, by age in women aged between 14 and 50 (N = 32,693). Although attendance rates for Chlamydia screening in Sweden are high (J. Dillner, personal communication), we anticipate that the very young (<17) and older women (>35) who attend elective screening are more likely to be at higher risk. We accommodated this in the model by specifying that a higher proportion of the high than the low sexual activity groups attended Chlamydia screening.

### Estimation of Progression and Clearance Rates for CIN-type Lesions

Six type-specific HPV progression and clearance rates of CIN1+ lesions were estimated from longitudinal data gathered as part of a large prospective randomised controlled trial of HPV testing and screening in Sweden (Swedescreen, see *Parameterisation and Data*). These rates were inferred from a corresponding vector of cumulative risks, derived from the observed 6-, 12- and 24-month proportions of viral clearance or disease progression among women with high-risk HPV infection. These rates were:

*μ*_1_: the rate of progression from initial HPV infection to C(G)IN1- type lesions

*μ*_2_: the rate of progression from C(G)IN1 to C(G)IN2 - type lesions

*μ*_3_: the rate of progression from C(G)IN2 to C(G)IN3 - type lesions

*μ*_4_: the rate of progression from CIN3 or CGIN3 - type lesions to squamous cell carcinoma or adenocarcinoma respectively

*X*_2_: the rate of clearance of C(G)IN1- type lesions

*X*_3_: the rate of clearance of C(G)IN2 and C(G)IN3– type lesions

**Figure 3:**
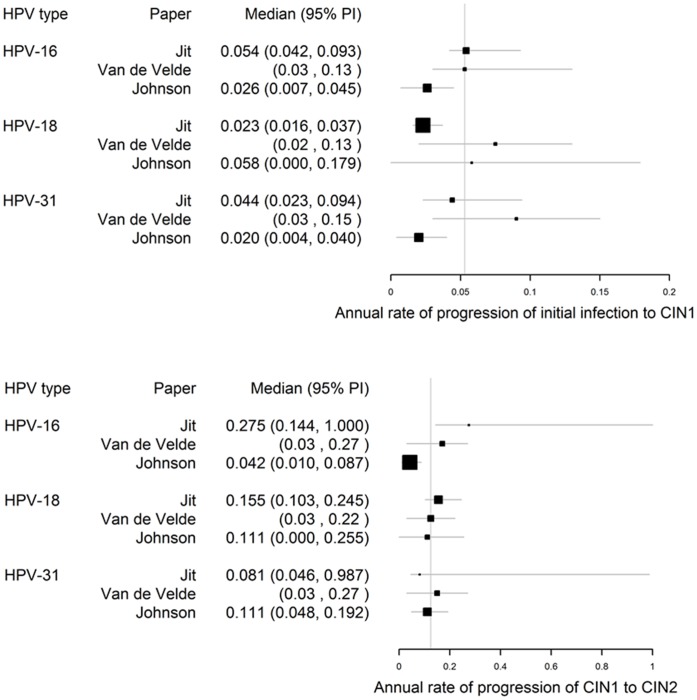
Estimated rates of progression to disease. (A) The estimated rates of progression of infection with HPV-16, HPV-18 and other high-risk (OHR) HPV types to CIN1-type lesions. We compare the rates estimated from the longitudinal Swedescreen data with the model-based estimates of Jit et al. [5] and Van de Velde et al. [21]. (B) The estimated rates of progression of CIN1-type lesions attributable to HPV-16, HPV-18 and other high-risk (OHR) HPV types to CIN2-type lesions. We compare the rates estimated from the longitudinal Swedescreen data with the model-based estimates of Jit et al. [5] and Van de Velde et al. [21].

These parameters were informed by a corresponding vector 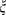 of cumulative risks, as derived from the longitudinal data of HPV-related disease progression among women tested positive for high-risk HPV infection at the baseline of the Swedescreen trial. The vector of cumulative risks is defined as follows:
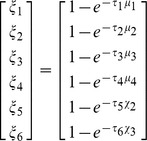



where


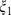
: the cumulative proportion of women with a high-risk HPV infection and normal cytology at baseline who were found to have borderline or mildly abnormal cytology after 24 months (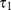 = 2 years)


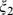
: the cumulative proportion of women with borderline or mildly abnormal cytology (CIN1) for the first time within the study, who were diagnosed with CIN2-type lesions after 6 months of follow-up (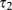 = 0.5 years)


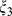
: the cumulative proportion of women with CIN2-type lesions for the first time within the study who were diagnosed with CIN3-type lesions after 12 months (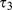 = 1 year)


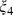
: the cumulative proportion of women with CIN3-type lesions for the first time within the study who were diagnosed with cancer after 24 months (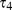 = 2 years)


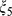
: the cumulative proportion of women with a high-risk HPV infection at baseline and borderline or mildly abnormal cytology (CIN1) for the first time within the study, who were found to have normal cytology after 12 months (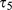 = 1 year)


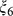
: the cumulative proportion of women with advanced CIN2 or CIN3-type lesions for the first time within the study, who were found to have normal cytology after 12 months (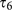 = 1 year)

**Figure 4:**
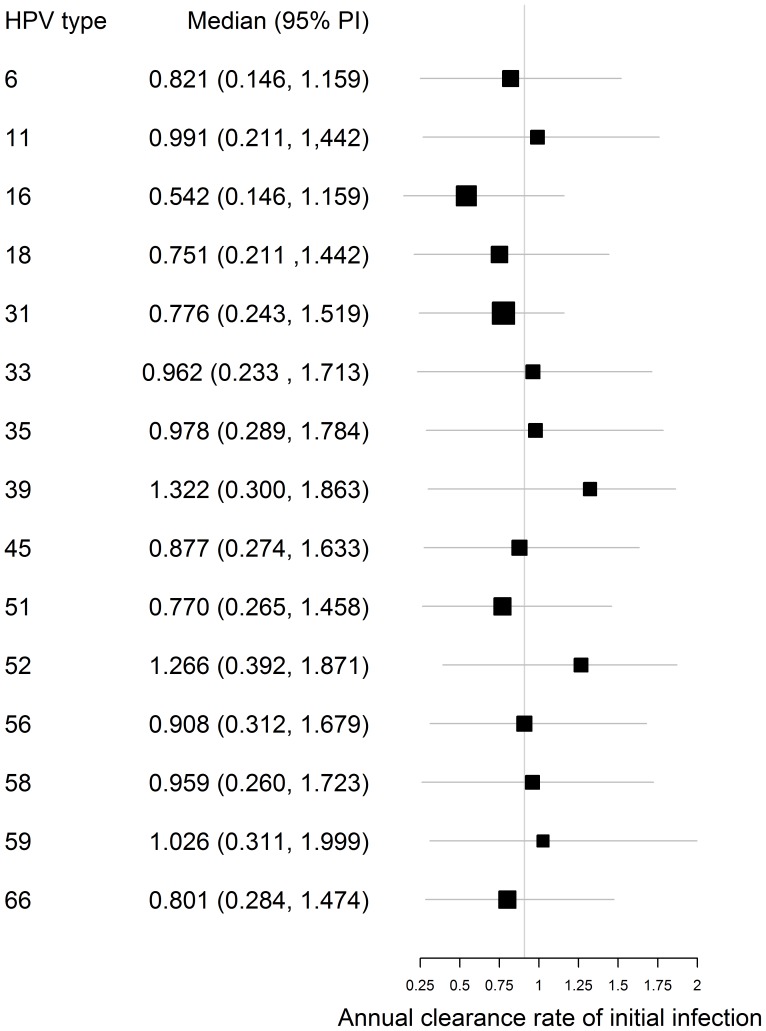
Comparison of the estimated annual rates of clearance of initial infection between 13 high-risk HPV types and HPV-6 and -11. The vertical line signifies the median of the median rates for all fifteen types; horizontal bars represent the 95% posterior intervals.

As outlined in Bogaards et al. [28], development of undiagnosed high-grade lesion among women with abnormal cytology at baseline is offset by the belated diagnosis of lesions that were already present at baseline, so the overall effect on the estimation of rates of progression to higher-grade lesion is very small.

There is an art to deciding after what time lag 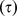 each parameter should be estimated: not so long that the disease has progressed by two or more stages but not so short that pending progression or clearance has not yet taken place. We made several estimates, corresponding to a few time points, estimating rates from each of them. The value for 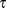 was chosen to be the shortest evaluated which was greater than 1/(its estimated progression rate). A bootstrap confidence interval (10,000 replications) was estimated for each progression and clearance rate.

### Assumptions

One limitation of the Swedescreen data was that each woman received only one HPV test as part of the Swedescreen trial – at baseline. As a consequence of this, it was necessary to assume that her history of infection and disease progression was attributable to the HPV type(s) that were identified at her baseline HPV test. Also, since it was not therefore possible to demonstrate clearance of infection, we assumed that a reversion of abnormal to normal cytology was an indicator of clearance of HPV.

Within the Swedescreen study, histological tests were only conducted if the results of a cytological test were positive. To paint a fuller picture of disease progression, we decided to include both cytological and histological data in our estimation of rates from longitudinal data. To do so, we considered equivalence between the grades shown in Table 2.

**Figure 5:**
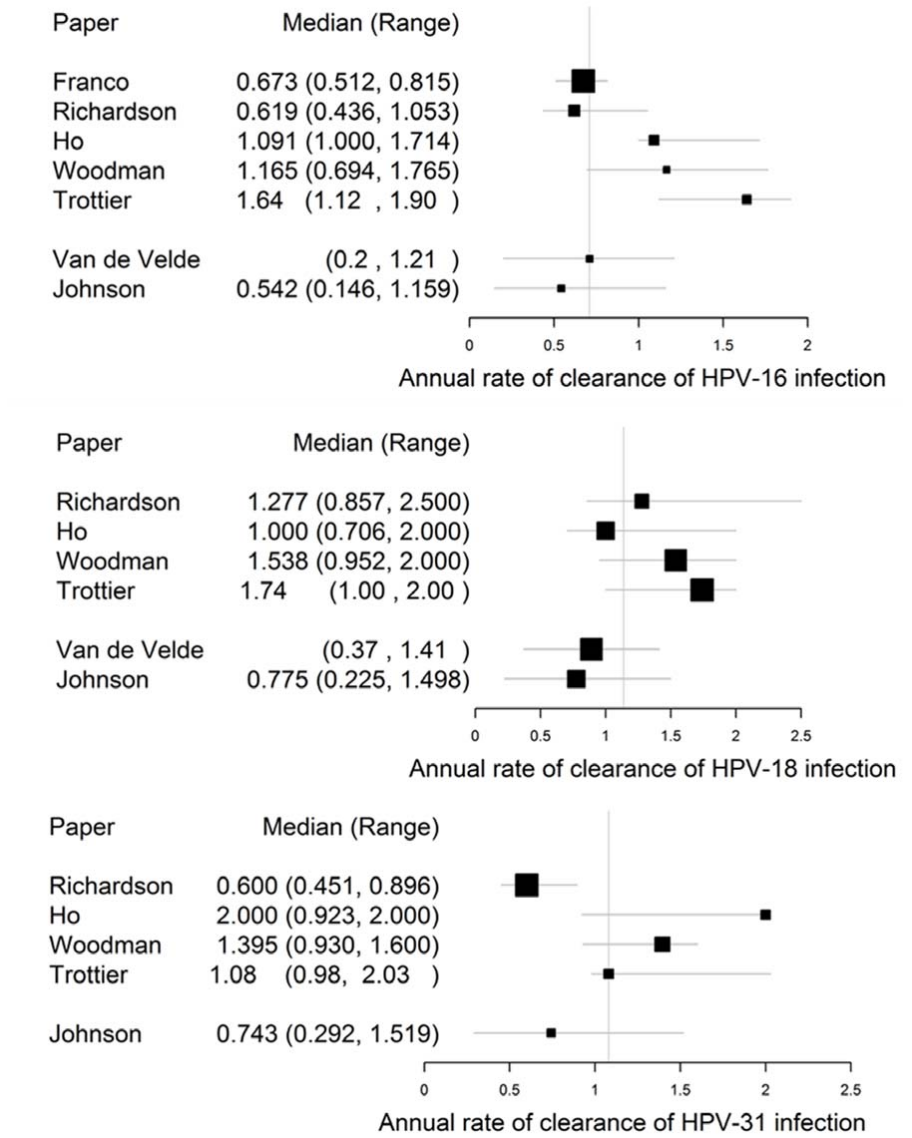
Annual rates of clearance of HPV infection. (A) Comparison of the estimated annual rates of clearance of initial HPV-16 infection in this and six further studies. The vertical line signifies the median of the median values of all seven studies. (B) Comparison of the estimated annual rates of clearance of initial HPV-18 infection in this and five further studies. The vertical line signifies the median of the median values of all six studies. (C) Comparison of the estimated annual rates of clearance of initial HPV-31 infection in four further studies. The vertical line signifies the median of the median values of all five studies.

In cases of a cancer diagnosis, we always relied on the definition from the histological test.

We assumed that development of disease was attributable to a hierarchy of HPV types: if a woman was infected with HPV-16 this was considered the cause of any cytological abnormalities; if she was infected with HPV-18 but not HPV-16, HPV-18 was considered the cause. To estimate rates for the other high-risk types (OHR) independently, we considered all women who tested positive for the type in question and negative for -16 and -18. This meant that we included in the estimation of OHR type progression and clearance, women who were co-infected with a number of OHR types. This was deemed necessary since many of the rarer types were only found in cases of co-infection with several OHR types.

**Table 4:** The estimated rates of transmissibility for thirteen high-risk HPV types and HPV-6 and -11.

HPV type	Median	95% posterior interval	
		Lower	Upper
6	0.755	0.295	1.000
11	0.741	0.256	1.000
16	0.718	0.286	1.000
18	0.736	0.292	1.000
31	0.743	0.292	1.000
33	0.752	0.268	1.000
35	0.746	0.264	1.000
39	0.755	0.233	1.000
45	0.739	0.279	1.000
51	0.755	0.233	1.000
52	0.745	0.317	1.000
56	0.743	0.253	1.000
58	0.758	0.311	1.000
59	0.745	0.252	1.000
66	0.757	0.275	1.000

Results presented are the median and 95% posterior interval.

In order to increase the effective sample size of women progressing from one state to another, we considered the first occurrence of a certain histological state for each woman to be her ‘baseline’ test at that level. For example, to estimate the rate of progression from CIN2 to CIN3 (*μ*_3_), we considered not only the women who were found to have CIN2 lesions at the baseline of the Swedescreen study, but all women who were found to have CIN2 lesions for the first time within the study; essentially the clock starts ticking as soon as her CIN2 diagnosis is made.

### Estimation of Further HPV-related Rates (HPV Transmissibility, Clearance of Initial Infection and Waning Immunity)

Type-specific estimates of the per-partnership transmissibility 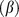, the annual waning resistance of natural immunity 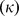 and the annual cumulative clearance rate of initial infection 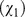 were estimated by fitting the deterministic model to two cross-sectional datasets, namely i) age- and type-specific prevalence of both high- and low-risk HPV types in men and women, aged 14–50, presenting for Chlamydia screening 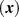 and ii) age- and type-specific prevalence of progressive cyto−/histological states in women in the intervention arm of the Swedescreen trial 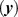. In the first instance, the progression and clearance rates estimated from the longitudinal data were also allowed to vary within prior ranges defined by their estimated bootstrap confidence intervals (see *Supplementary Material*) but since it was found that the resultant posterior distributions were not noticeably responsive to these parameters, the rates were fixed at their median values. The following Bayesian model framework was used:

**Figure 6:**
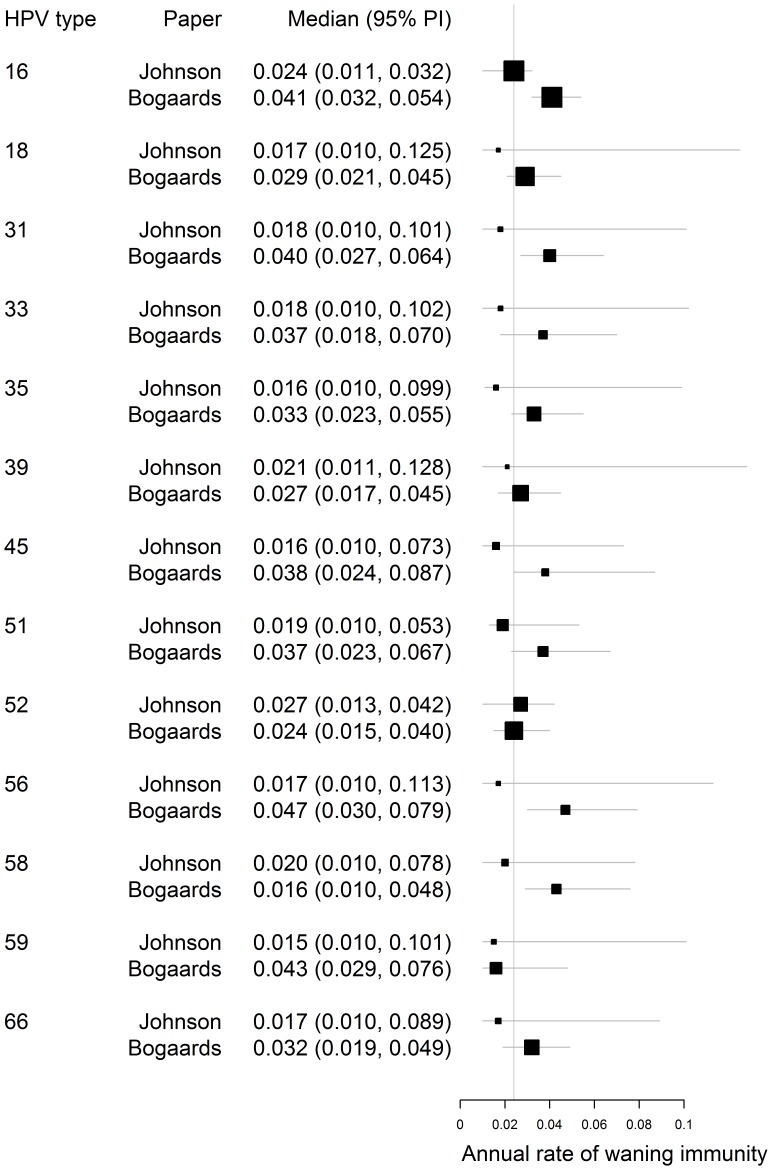
Comparison of the estimated annual rates of waning immunity for 13 high-risk types of HPV with those estimated by Bogaards et al. [**22**] **.** The vertical line signifies the average of the posterior median estimates across all types in both studies.



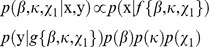



The likelihood function for the Chlamydia screening programme HPV prevalence data, 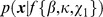 is a straightforward multivariate Binomial with success probability 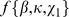, whereas the likelihood function for the Swedescreen data for prevalence by stage, 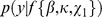 is a multivariate Multinomial with success probability 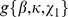.

The function 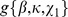 is simply the probability function
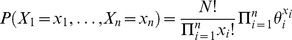



where

*X*_1_,*X*_2_,…,*X_n_*. are a set of random variates


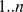
 are non-negative integers such that



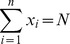
and 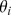 are constants with 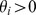
and



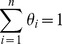



Thus we are able to jointly consider the ‘success’ probability of being in the susceptible state, the initial infection state, the CIN1 state and so forth.

Each of the 13 high-risk HPV types was modelled separately. Estimates of the three natural history parameters were derived from posterior distributions of the Markov Chain Monte Carlo (MCMC) chains (chain length  = 20,000) using the Metropolis-Hastings algorithm [29]. The prior distribution for per-partnership transmissibility, 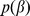, was uniform on 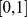, for annual rate of clearance of initial infection, 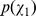, was uniform on 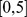 and the inverse of 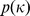, the duration of infection-induced immunity in years, was uniform on 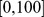. Since a preliminary analysis attempting to infer the rate of loss of infection-induced immunity, 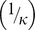, led to a posterior heavily skewed towards zero, it was decided to estimate the duration rather than the rate. This tendency of the rate of loss of immunity towards zero is suggestive of an underlying SIR process but, in setting the prior for duration of immunity from 0 to 100 years, we still allow for the possibility of an SIRS model structure. This is consistent with the observation by Baussano et al. [30] that other modelling studies [20], [21], [31] have found it necessary to assume some measure of induced or long-lasting immunity in order to achieve a good model fit.

In each iteration of the algorithm, a proposal for 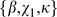 was sampled from a multivariate normal distribution with standard deviation 0.04 times the range of the corresponding prior density. The deterministic model was then run for this proposal set and the likelihood calculated as detailed in the main body of the paper. The proposal parameter set was then accepted with a probability determined by the ratio of its posterior density to that of the previous set.

For each type the algorithm was run for a chain of length n = 20,000. Visual inspection confirmed that the Metropolis-Hastings algorithm converged swiftly to the posterior distribution and parameter estimates were made rejecting a ‘burn-in’ period of 100 iterations. Examples of the MCMC traces are shown in Figures S1 and S2 for HPV-16 and HPV-45 respectively. We report 95% posterior intervals for the posterior distributions of all MCMC-estimated parameters. These are obtained from the 2.5^th^ and 97.5^th^ percentiles of all iterates after the burn-in period.

## Results

The observed age-dependent prevalence of high-risk HPV infection was accurately replicated by the appropriate type-specific model for each type (see Figure 2). In general, a good fit to the data was observed, with the model able to replicate the rapid acquisition of markers, the peak in prevalence in women in their twenties, and the decline thereafter. In the cases of HPV-33, -35, -45 and -58, the models over-estimated the prevalence of infection with respect to the data. This was not due to a failure of the Markov chain to converge (see *Supplementary Material*; further data available on request). The prevalence of HPV-16 infection in women taking part in the Swedescreen trial was higher than those of the same age (32–38) attending Chlamydia testing, HPV-18 and HPV-33 were observed at closely comparable levels in both studies but all other high-risk types were less prevalent in the former group. This disparity probably represents the higher representation of highly sexually active women in the voluntarily attending Chlamydia screening in their mid-thirties.

### Estimation of Progression and Clearance Rates of CIN-type Lesions

The median estimated rates of progression and clearance of CIN1-type lesions in four high-risk HPV types, each with a 95% adjusted bootstrap confidence interval, are given in Table 3. Due to a paucity of data on some of the rarer high-risk types, it was not possible to estimate each progression/clearance rate for each of the 13 high-risk types. Thus we estimated rates for ‘other’ high-risk (OHR) types by pooling data. To facilitate comparison with other studies, the grouping OHR excludes only HPV-16 and HPV-18, even though we had been able to estimate some rates for types -31 and -33.

The progression and clearance rates did not differ significantly between HPV types. However, HPV-16 appeared to progress from initial infection to CIN1- type lesions at an annual rate of 0.026 (0.007–0.045), somewhat more slowly than HPV-18 which had an annual rate of 0.058 (0–0.179) but at a similar rate to other high-risk types (OHR) which had an annual rate of 0.02 (0.004–0.04).

Figure 3 shows the comparison of our data-derived rates of progression of CIN1- and CIN2-type lesions with the estimates of Jit et al. [5] and Van de Velde et al. [21].

### Estimation of Clearance Rate of Initial Infection

The rate of clearance of type-specific initial HPV infection was well characterised by the posterior distribution of the MCMC chain, with a median posterior/prior ratio of 0.320 across all HPV types. The median values for the rate of clearance of initial infection did not differ significantly between types (see Figure 4). Our estimated clearance rates tended to be lower than the estimates of Trottier et al. [16] from data on HPV infection among women in São Paulo, Brazil but were in line with other cohort studies for the types where estimates exist (see Figure 5).

Although few estimates have been made of the annual clearance rates of some of the rarer high-risk HPV types, more cohort studies have concentrated on estimates for HPV-16, -18 and the grouped other high-risk (OHR) types. In Figure 5 we compare our estimates with those of four further cohort studies and with the model-based estimates of Van de Velde et al. [21]. The confidence intervals of our estimates overlap with those of the other studies.

### Estimation of Transmissibility

The median estimated rates of transmissibility in the thirteen high-risk HPV types, each with a 95% adjusted bootstrap confidence interval, are given in Table 4. The median estimates were not significantly different, ranging from 0.718 (0.286–1.000) for HPV-16 to 0.758 (0.311–1.000) for HPV-58. In fact, the posteria of all thirteen transmission probabilities were skewed towards the inherent upper bound of 1.

### Estimation of Rate of Waning Immunity

The estimated rates of waning natural immunity (or resistance to subsequent infection) are shown in Figure 6. Our estimates do not differ significantly from those of Bogaards et al. [22] but the posterior median values are consistently lower, corresponding to 5-year cumulative proportions of clearance of 6%–12% across the 13 types (annual rates of 0.015–0.027). HPV-16 was among those with the highest median estimates with an estimated 5-year cumulative proportion of clearance of 11.4% (5.4%–15.0%). Such low rates of waning natural immunity imply that the protection conferred by infection with a certain HPV type might indeed be, at least for some, life-long; an assumption held in other modelling studies [13], [32].

## Discussion

We have made estimates for the transmissibility, clearance and progression rates and rates of waning natural immunity for 13 high-risk types of HPV by using Swedish behavioural and epidemiological data to fit an ensemble of deterministic models of HPV transmission and progression. The datasets used included the ‘*Sex in Sweden’* sexual behaviour survey, longitudinal data on HPV prevalence and clinical disease progression from the randomised controlled trial of HPV testing, ‘*Swedescreen’*, and HPV type-specific prevalence data gathered as part of the Swedish voluntary Chlamydia screening programme. The analysis was formed of two stages. Firstly, rates were estimated for the stepwise progression of different HPV types to disease of increasing clinical severity and of clearance of different grades of late-stage infection. Secondly, these estimated rates were incorporated into a monotype SIRS-structured deterministic model of the transmission and progression of HPV. This model was then fitted to HPV prevalence data using a Markov Chain Monte Carlo (MCMC) routine, in order to estimate the transmissibility, rate of clearance of initial infection and rate of waning natural immunity of each type in turn.

It was observed that the models over-estimated the observed prevalence of four of the high-risk types. We suggest that this is an artefact of modelling the HPV types independently. Three of these types (-33, -35 and -58) are, in fact, three of the most closely phylogenetically-related to HPV-16; the most prevalent type. HPV-45 is the most closely related to the second most prevalent type, HPV-18. It is feasible that this similarity leads to cross-immunity between the types, effectively reducing the number of young women susceptible to infection with types -33, -35, -45 and -58.

We found variation in the rates of clearance of initial infection to be a very informative parameter in the fitting of type-specific models of HPV prevalence to data, indicating that this may be an important determinant in the differing prevalences between types. The lowest rates of clearance of initial infection were observed in HPV-16, -18 and -31, the most prevalent of the high-risk HPV types. This would again support the theory that higher prevalence is associated with persistence of infection, though we would agree with Bogaards et al. [22] that this does not preclude a role for transmissibility and immunity.

Our estimates of transmissibility did not differ significantly between types although there remained a large degree of uncertainty. Our inferred values were in line with those estimated in previous studies: the median per-partnership transmissibility of HPV-16 was estimated to be 0.718 with a 95% posterior interval of 0.286–0.999) compared with 0.8 estimated by Hughes et al. [11]. However, values for all types were skewed towards the maximum limit of 1. We suggest that this may be attributable either to an underestimate in the sexual activity data or to the assumption of proportionate random mixing by age and sexual activity group, or to a combination of the two. Barnabas et al. [13] had a similar finding, noting that survey data will tend to underestimate sensitive sexual behaviour such as partner change rates. This study has the further complication that the sexual activity data used, although well matched to the study population in geographical and demographical terms, predates the epidemiological data used for fitting (and the time period of modelling interest) by at least ten years. It is reasonable to suppose that sexual behaviour trends in the intervening decade may well have given rise to a higher HPV prevalence than that contemporaneous with the sexual activity data, requiring an increase in the inferred transmissibility to compensate. Alternatively, the inferred transmission probabilities may have been pushed higher by assuming completely proportionate mixing. In fact this is a very strong assumption: age-dependent mixing in particular is known to be assortative and concentration of transmission in certain age groups may reduce the transmission probabilities needed to fit the observed data.

The rate of waning immunity was estimated to be very low; the 5-year cumulative proportions of clearance varied between 6%–12% across the 13 types. This would imply that few women become susceptible to re-infection with any given HPV type and that the observed decrease in the force of infection in the early twenties is more likely to be due to immunity than to decreased sexual activity. Indeed, our estimates of the rate of waning immunity would imply that the protection conferred by infection with HPV is effectively close to life-long.

We have also further contributed to the literature estimating the rates of progression and clearance of clinical lesions due to HPV, extending the field by deriving type-specific estimates of progression rates up to CIN3-grade lesions for type -31 and 33 and clearance thereof. Our results implied that the rate of progression of other high-risk types from CIN1 to higher grade lesions was significantly lower than for HPV-16 and HPV-18, indicating that their less prominent role in the cause of cervical cancer may be due not solely to lower prevalence but also to an inherently lower oncogenicity. Our estimates are similar to others in the literature although they are subject to a large degree of uncertainty, highlighting the benefit that would be derived from pooling data on disease progression from *Swedescreen* with those collected as part of other studies elsewhere. Such collation would increase the power of the analysis, ensuring more certainty in the inferred rates.

Although this analysis provides some useful new insight into HPV-related parameters, it has some limitations. Firstly, the nature of deterministic compartmental models means that it is not possible to consider diversity in individual-level behaviour. For example, we estimate here the per-partnership transmissibility of HPV infection. In order to derive from this a per-act transmissibility requires us to assume an ‘average’ partnership between a susceptible and an infected partner. Also, we assume that all women have an equivalent approach to screening and subsequent treatment. Such an assumption belies the true underlying heterogeneity; in fact, it is those women who do not attend screening who are more likely to go on to develop high-grade clinical lesions and cervical cancer. In considering the more subtle questions policy-makers face in terms of vaccination and screening programmes it is common for complex individual-based models to be used. However, the fitting of these models is similarly complex due to the long run times and inherent stochasticity of these models. The type-specific estimates we have made here for HPV transmissibility and rates of progression and clearance will facilitate the parameterisation of such individual-based models by, for example, providing prior ranges for natural history parameters. These models will then, in turn, allow a more thorough understanding of the complex natural history of HPV infection and its implications for the efficacy of vaccine and screening programmes in this new era of HPV prevention.

## Supporting Information

Figure S1 **MCMC traces (left) and posterior distributions (right) for estimation of HPV-16 parameters.** The MCMC chains (n = 20,000) and posterior distribution density are shown for, in order, the transmissibility (*β*), cumulative clearance rate of initial infection (*X*_1_) and rate of waning of natural immunity *X*_1_..*X*_3_ for HPV-16. The fourth row shows the trace and distribution of the log-likelihood. The figures show a rapid convergence.
(TIFF)

Figure S2
**MCMC traces (left) and posterior distributions (right) for estimation of HPV-45 parameters.** The MCMC chains (n = 20,000) and posterior distribution density are shown for, in order, the transmissibility 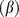, cumulative clearance rate of initial infection 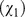 and rate of waning of natural immunity 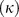 for HPV-45. The fourth row shows the trace and distribution of the log-likelihood. The figures show a rapid convergence.(TIFF)

Supplementary Material S1 (DOCX)
