# Supplementary figures and images for: Inference of Type-Specific HPV Transmissibility, Progression and Clearance Rates: A Mathematical Modelling Approach
Source: PLoS One. 2012 Nov 21;7(11):e49614. doi: 10.1371/journal.pone.0049614 (PMC3504161; doi:10.1371/journal.pone.0049614)

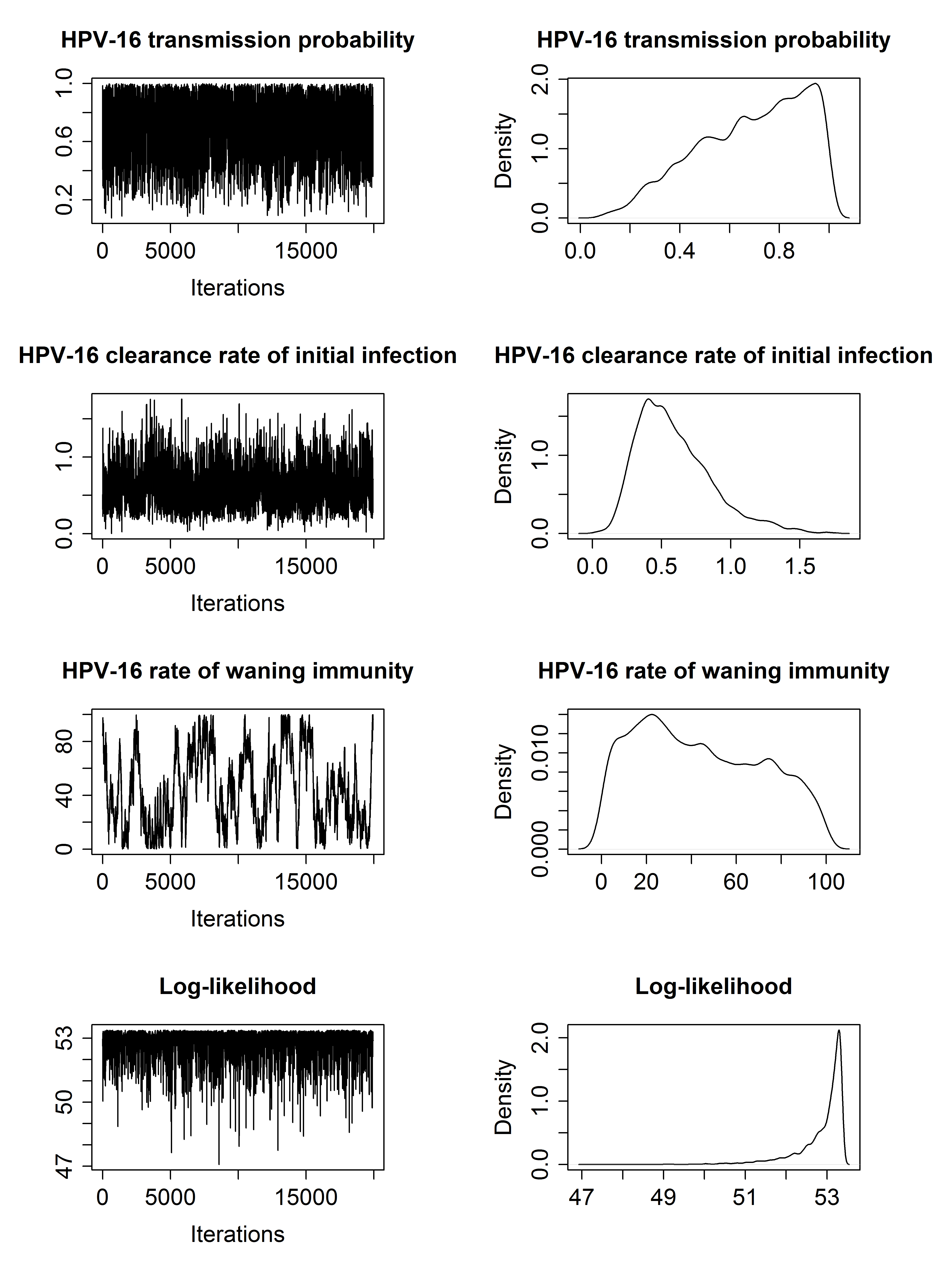

Supplement: Figure S1 — MCMC traces (left) and posterior distributions (right) for estimation of HPV-16 parameters. The MCMC chains (n = 20,000) and posterior distribution density are shown for, in order, the transmissibility (β), cumulative clearance rate of initial infection (X 1) and rate of waning of natural immunity X 1..X 3 for HPV-16. The fourth row shows the trace and distribution of the log-likelihood. The figures show a rapid convergence. (TIFF) [file pone.0049614.s001.tiff]

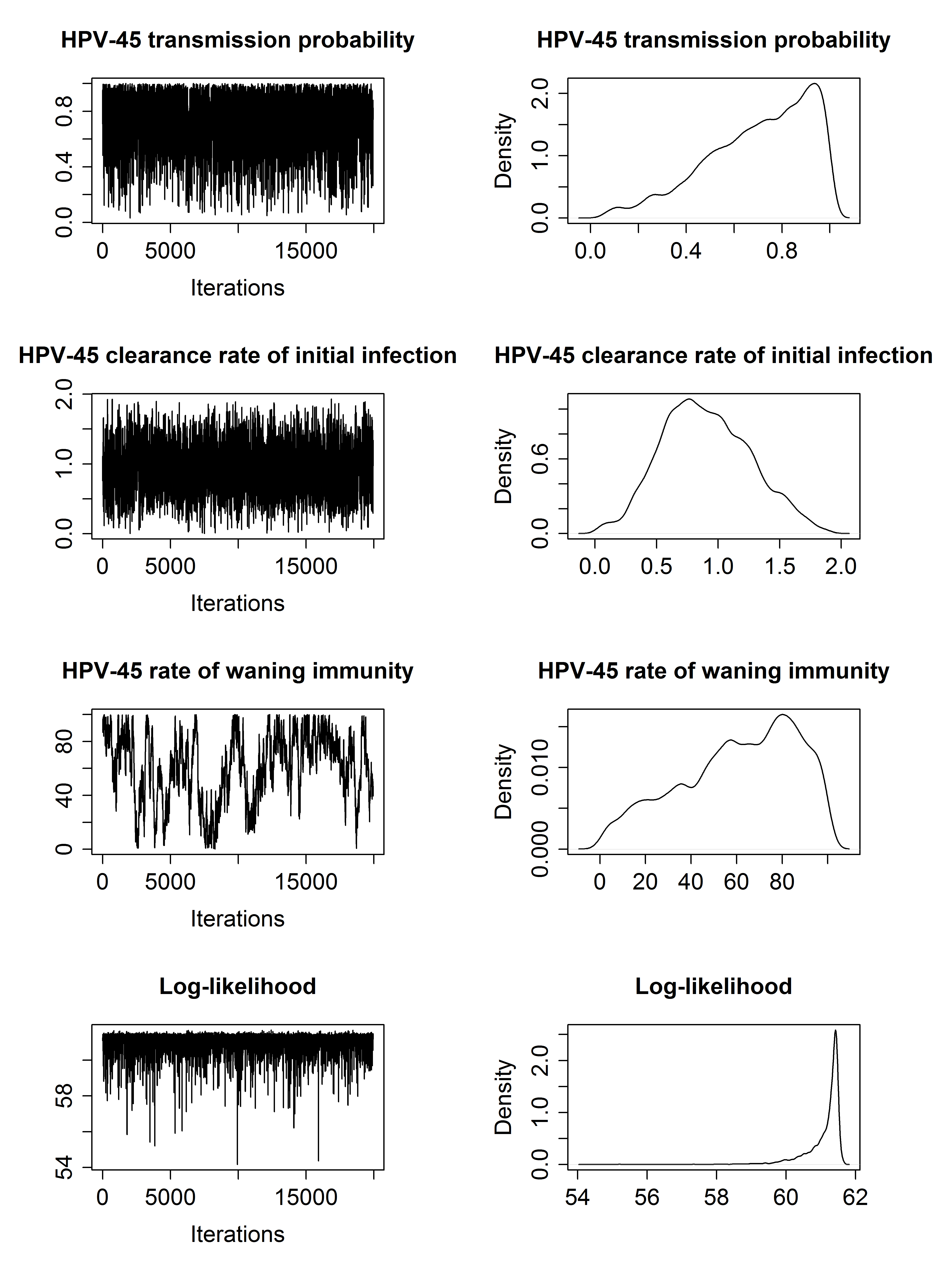

Supplement: Figure S2 — MCMC traces (left) and posterior distributions (right) for estimation of HPV-45 parameters. The MCMC chains (n = 20,000) and posterior distribution density are shown for, in order, the transmissibility , cumulative clearance rate of initial infection and rate of waning of natural immunity for HPV-45. The fourth row shows the trace and distribution of the log-likelihood. The figures show a rapid convergence. (TIFF) [file pone.0049614.s002.tiff]
