# Supplementary material for: Inference of Type-Specific HPV Transmissibility, Progression and Clearance Rates: A Mathematical Modelling Approach
Source: PLoS One. 2012 Nov 21;7(11):e49614. doi: 10.1371/journal.pone.0049614 (PMC3504161; doi:10.1371/journal.pone.0049614)
Supplement: Supplementary Material S1 — (DOCX) [file pone.0049614.s003.docx]

# Supporting information

## HPV transmission model equations

**Females**

$$\begin{matrix} \frac{\partial S_{1,i}(a,t)}{\partial a}+\frac{\partial S_{1,i}(a,t)}{\partial t}=\left(1-\left(\omega+\lambda_{1,i}\left(a,t\right)\right)\right)S_{1,i}(a,t)+\kappa R_{1,i}\left(a,t\right)+\alpha N_{1} \\ \frac{\partial I_{1,i}^{1}(a,t)}{\partial a}+\frac{\partial I_{1,i}^{1}(a,t)}{\partial t}=\left(1-\left(\omega+\mu_{1}+\chi_{1}\right)\right)I_{1,i}^{1}(a,t)+\lambda_{1,i}\left(a,t\right)S_{1,i}(a,t) \\ \frac{\partial I_{1,i}^{2}(a,t)}{\partial a}+\frac{\partial I_{1,i}^{2}(a,t)}{\partial t}=\left(1-\left(\omega+\mu_{2}+\chi_{2}\right)\right)I_{1,i}^{2}(a,t)+\mu_{1}I_{1,i}^{1}(a,t) \\ \frac{\partial I_{1,i}^{3}(a,t)}{\partial a}+\frac{\partial I_{1,i}^{3}(a,t)}{\partial t}=\left(1-\left(\omega+\mu_{3}+\chi_{3}+\pi\right)\right)I_{1,i}^{3}(a,t)+\mu_{2}I_{1,i}^{2}(a,t) \\ \frac{\partial I_{1,i}^{4}(a,t)}{\partial a}+\frac{\partial I_{1,i}^{4}(a,t)}{\partial t}=\left(1-\left(\omega+\mu_{4}+\chi_{3}+\pi\right)\right)I_{1,i}^{4}(a,t)+\mu_{3}I_{1,i}^{3}(a,t) \\ \frac{\partial I_{1,i}^{5}(a,t)}{\partial a}+\frac{\partial I_{1,i}^{5}(a,t)}{\partial t}=\left(1-\left(\omega+\pi+\upsilon\right)\right)I_{1,i}^{5}(a,t)+\mu_{4}I_{1,i}^{4}(a,t) \\ \frac{\partial R_{i}(a,t)}{\partial a}+\frac{\partial R_{i}(a,t)}{\partial t}=\left(1-\left(\omega+\kappa\right)\right)R_{i}(a,t)+\pi\left(I_{1,i}^{3}(a,t)+I_{1,i}^{4}(a,t)+I_{1,i}^{5}(a,t)\right)+\chi_{1}I_{1,i}^{1}((a,t)+\chi_{2}I_{1,i}^{2}(a,t)+\chi_{3}\left(I_{1,i}^{3}(a,t)+I_{1,i}^{4}(a,t)\right) \end{matrix}$$

**Males**

$$\begin{matrix} \frac{\partial S_{2,i}(a,t)}{\partial a}+\frac{\partial S_{2,i}(a,t)}{\partial t}=\left(1-\left(\omega+\lambda_{2,i}\left(a,t\right)\right)\right)S_{2,i}(a,t)+\kappa R_{2,i}\left(a,t\right)+\alpha N_{1} \\ \frac{\partial I_{2,i}(a,t)}{\partial a}+\frac{\partial I_{2,i}(a,t)}{\partial t}=\left(1-\left(\omega+\chi_{1}\right)\right)I_{2,i}(a,t)+\lambda_{2,i}\left(a,t\right)S_{2,i}(a,t) \\ \frac{\partial R_{2,i}(a,t)}{\partial a}+\frac{\partial R_{2,i}(a,t)}{\partial t}=\left(1-\left(\omega+\kappa\right)\right)R_{2,i}(a,t)+\chi_{1}I_{2,i}(a,t) \end{matrix}$$

where

$S_{g,i}(a,t)$: persons of gender, *g* , and age, *a*, who are susceptible to HPV type at time *t*

$I_{g,i}^{1}(a,t)$: persons of gender, *g* , and age, *a* , who are infected with initial HPV type at time *t*

$I_{1,i}^{2}(a,t)$: women of gender, *g* , and age, *a*, who have CIN1-type lesions attributable to HPV type at time *t*

$I_{1,i}^{3}(a,t)$: women of gender, *g* , and age, *a*, who have CIN2-type lesions attributable to HPV type at time *t*

$I_{1,i}^{4}(a,t)$: women of gender, *g* , and age, *a*, who have CIN3-type lesions attributable to HPV type at time *t*

$I_{1,i}^{5}(a,t)$: women of gender, *g* , and age, *a*, who have cancer attributable to HPV type at time *t*

$R_{g,i}(a,t)$: persons of gender, *g* , and age, *a*, who are resistant to HPV type at time *t*

*$N_{g}$*: population size of gender, *g*

$\lambda_{g,i}$: force of infection acting on persons of gender, *g* , and sexual activity group, *i*

$\kappa$: annual rate of loss of naturally-acquired immunity

$\mu_{1}$: annual rate of progression from initial infection to CIN1-type lesions

$\mu_{2}$: annual rate of progression from CIN1-type lesions to CIN2-type lesions

$\mu_{3}$: annual rate of progression from CIN2-type lesions to CIN3-type lesions

$\mu_{4}$: annual rate of progression from CIN3-type lesions to cancer

$\chi_{1}$: annual rate of clearance of initial infection

$\chi_{2}$: annual rate of clearance of CIN1-type lesions

$\chi_{3}$: annual rate of clearance of CIN2-type lesions

$\pi$: combined rate for screening and subsequent treatment of lesions

$\alpha$: birth rate

$\omega$ : natural-cause mortality

$\upsilon$: rate or mortality due to cervical cancer
